# A Reply to Dr. Guernsey’s Defence

**Published:** 1890-08

**Authors:** Edmund Carleton

**Affiliations:** 53 West Forty-fifth St., New York


### A REPLY TO DR. GUERNSEY'S DEFENCE.

53 West Forty-fifth St., New York, July 10th, 1890.

Editor Homœopathic Physician:—Your note received, calling attention to Dr. Egbert Guernsey's communication in this month's number of your journal.

I have no quarrel with Dr. Guernsey, and cannot follow him into the arena of personalities. My letter to you, which appeared in the June number, simply defined my position, and attacked nobody.

A book is kept at the hospital, in which members of the Medical Board sign their names whenever they visit their wards. The inclosed is a transcript from that book for the year 1889. We attend two months at a time, and then rest four months.

According to the record, I made twelve visits. If you wish to have a history of previous years, please signify.

EDMUND CARLETON.

[The record shows that Dr. Carleton made twelve visits, while Dr. Guernsey made one. Further comment seems unnecessary.—EDS.]

